# Diabetesmanagement im Krankenhaus (Update 2026)

**DOI:** 10.1007/s00508-026-02717-9

**Published:** 2026-04-30

**Authors:** Julia K. Mader, Felix Aberer, Johanna M. Brix, Bernhard Föger, Daniel A. Hochfellner, Antonia-Therese Kietaibl, Bernhard Ludvik, Michael Resl, Claudia Ress-Winkel, Lars Stechemesser, Alexander Vonbank, Harald Sourij, Martin Clodi

**Affiliations:** 1 Universitätsklinik für Innere Medizin, Klinische Abteilung für Endokrinologie und Diabetologie, Medizinische Universität Graz, Graz, Österreich; 2 1. Medizinische Abteilung mit Diabetologie, Endokrinologie und Nephrologie, Klinik Landstraße, Wien, Österreich; 3 Abteilung für Innere Medizin, Fachklinik Schwaben, Bad Mergentheim, Deutschland; 4 5. Medizinische Abteilung mit Endokrinologie, Rheumatologie und Akutgeriatrie mit Ambulanz, Klinik Ottakring, Wien, Österreich; 5 Abteilung für Innere Medizin, Konventhospital der Barmherzigen Brüder Linz, Klinisches Forschungsinstitut für kardiovaskuläre und metabole Erkrankungen, Johannes Kepler Universität Linz, Linz, Österreich; 6 Universitätsklinik für Innere Medizin 1, Medizinische Universität Innsbruck, Innsbruck, Österreich; 7 Universitätsklinik für Innere Medizin I mit Gastroenterologie-Hepatologie, Nephrologie, Diabetologie und Stoffwechselerkrankungen, Universitätsklinikum der Paracelsus Medizinischen Privatuniversität, Salzburg, Österreich; 8 Abteilung für Innere Medizin I, akademisches Lehrkrankenhaus Feldkirch, Vorarlberg, Österreich; 9 VIVIT-Institut, Feldkirch, Österreich; 10 1. Medizinische Abteilung, Klinik Favoriten, Wien, Österreich

**Keywords:** Diabetes mellitus, Stationäre Versorgung, Hyperglykämie, Inpatient care, Hyperglycemia, Hospital care, Diabetes mellitus

## Abstract

Dieses Positionspapier beinhaltet die Empfehlungen der Österreichischen Diabetes Gesellschaft zum Management von Patient*innen mit Diabetes mellitus während stationärer Aufenthalte und im perioperativen Setting, basierend auf aktueller Evidenz zu Glukosezielbereichen, Insulintherapie und Therapie mit oralen/injizierbaren Antidiabetika. Zusätzlich werden Spezialsituationen wie intravenöse Insulintherapie, begleitende Glukokortikoidtherapie sowie die Anwendung von Diabetestechnologie im stationären und perioperativen Bereich diskutiert.

## Abstract (en)

This position statement presents the recommendations of the Austrian Diabetes Association for the management of patients with diabetes during inpatient stay and in the perioperative setting. These recommendations are based on current evidence with respect to glucose targets, insulin therapy and treatment with oral/injectable antihyperglycemic agents during hospitalization and in perioperative setting. Additionally, it discusses special situations such as intravenous insulin therapy, concomitant therapy with glucocorticoids and use of diabetes technology during hospitalization and the perioperative setting.

## Prävalenz von Hyperglykämien im Krankenhaus

Epidemiologische Daten zeigen, dass – vereinbar mit der globalen Zunahme an Diabeteserkrankungen – auch die Anzahl an Patient*innen mit Diabetes und Hyperglykämien im Krankenhaus deutlich ansteigt, wobei die amerikanische Diabetesgesellschaft (ADA) eine Nüchternblutglukose über 140 mg/dl als Hyperglykämie definiert [1].

Die sog. Stresshyperglykämie beschreibt den Zustand erhöhter Blutglukosewerte bei akuten Erkrankungen und tritt als Folge von meist kurzfristigen metabolischen, inflammatorischen und hormonellen Dysregulationen auf. Die Stresshyperglykämie stellt in der Regel ein reversibles Begleitphänomen akuter Erkrankungen dar, persistiert jedoch häufig im Falle einer Demaskierung einer vorbestehenden Glukosetoleranzstörung. Unabhängig davon zeigte sich, dass die Stresshyperglykämie in verschiedensten Populationen einen potenteren Risikofaktor für gesundheitliche Komplikationen im Krankenhaus darstellt als die Hyperglykämie bei Patient*innen mit vorbekannter Diabeteserkrankung [2].

Unabhängig vom Vorliegen eines vorbekannten Diabetes mellitus wird die weltweite Prävalenz des Auftretens von Hyperglykämien bei hospitalisierten Patient*innen auf 20–40 % geschätzt [3, 4], wobei kritisch kranke Patient*innen auf Intensivstationen in bis zu 70 % Hyperglykämien aufweisen. Die Prävalenz hyperglykämischer Episoden während eines Krankenaufenthaltes korreliert wie auch die Diabetesprävalenz stark mit dem Alter der Patient*innen. So zeigte sich, dass über 75-Jährige mit einer 2,4-fach höheren Wahrscheinlichkeit mit einer Diabetesdiagnose aus dem Krankenhaus entlassen werden als eine Kontrollgruppe unter 65 Lebensjahren [5, 6]. Rezente Daten aus Österreich zeigten, dass, basierend auf einer HbA_1c_-Messung während des stationären Aufenthalts, bei mehr als der Hälfte der internistischen Patient*innen eine Dysglykämie (Prädiabetes oder Diabetes) vorlag. Davon hatten 27,8 % Diabetes und 23,7 % Prädiabetes. Bei 2,4 % handelte es sich um eine Erstdiagnose eines Diabetes. Die höchste Prävalenz fand sich in der Altersgruppe der 70- bis 79-Jährigen (36,8 % Diabetes, 24,8 % Prädiabetes) [7].

Perioperative Hyperglykämien (> 140 mg/dl) treten bei Menschen mit und ohne Diabetes mit 20–40 % bei allgemeinchirurgischen und 80–90 % bei kardiochirurgischen Patient*innen sehr häufig auf [8–12].

## Auswirkungen von Hyperglykämien im Krankenhaus

Hyperglykämien bei Patient*innen im Krankenhaus stellen sowohl auf Normalstationen als auch auf Intensivstationen einen erheblichen und unabhängigen Risikofaktor für erhöhte Mortalität und gesundheitliche Komplikationen wie Infektionen (z. B. Pneumonien) [13] oder Operationskomplikationen [14] dar. Eine Assoziation besteht einerseits mit der Höhe der Hyperglykämie bei Krankenhausaufnahme und andererseits mit der mittleren Glukose während des gesamten Krankenhausaufenthaltes [15–17].

Menschen mit Diabetes mellitus weisen ein prinzipiell erhöhtes Risiko für Infektionen bzw. für postoperative Infektionskomplikationen auf [18–24]. Die Art des operativen Eingriffs ist nicht nur für die präoperative Evaluierung, sondern auch für das postoperative Komplikationsrisiko entscheidend: Bei herzchirurgischen Eingriffen war das postoperative Infektionsrisiko bei Diabetes mellitus mit einer OR von 2,03 signifikant höher als bei den übrigen chirurgischen Eingriffen. Auch das Auftreten postoperativer kardiovaskulärer Komplikationen ist bei Patient*innen mit Diabetes mellitus Typ 2 nach kardialen Eingriffen signifikant erhöht [25]. Erhöhte präoperative Blutzuckerwerte sind ebenso mit einem erhöhten Risiko für postoperative Komplikationen assoziiert [26–28]. Daher sollte bei akuten Blutzuckerentgleisungen mit Hyperglykämien über 300 mg/dl eine elektive Operation postponiert werden, bis sich die Blutglukose zumindest für 1–4 h präoperativ im Zielbereich (zumindest < 180 mg/dl) befindet und Patient*innen metabolisch kompensiert sind [27, 29, 30].

Abgesehen von der Tatsache, dass hospitalisierte Patient*innen mit Hyperglykämien höhere Krankenhauskosten verursachen [31, 32], besteht auch ein höheres Risiko für längere Krankenhausaufenthalte [33, 34]. Zudem zeigte sich, dass Patient*innen mit Hyperglykämien während des stationären Aufenthalts häufiger eine poststationäre Rehabilitation und/oder einen Transfer in eine medizinisch betreute Wohneinrichtung (z. B. Pflegeheim) in Anspruch nehmen müssen [35].

Neben den ungünstigen Auswirkungen von Hyperglykämien im Krankenhaus selbst benötigen Menschen mit Diabetes mellitus aufgrund der mit dem Diabetes einhergehenden mikro- und makrovaskulären und neuropathischen Spätkomplikationen häufiger akute und geplante stationäre Aufnahmen [36, 37]. Darüber hinaus führen Diabetes-assoziierte Komplikationen wie hyperglykämisches Koma, diabetische Ketoazidose und iatrogene Hypoglykämie häufig zur Indikationsstellung einer akuten Krankenhauseinweisung [38]. Neben der Hyperglykämie stellte auch das HbA_1c_ bei Aufnahme einen unabhängigen Prädiktor für ungünstige Outcomes betreffend Krankenhausaufenthaltsdauer, Infektionen und Mortalität in unterschiedlichen Kollektiven dar [39–41].

## Blutglukosezielwerte und Blutglukosemessfrequenz bei hospitalisierten Patient*innen

### Kritisch kranke Patient*innen

- Bei anhaltender Hyperglykämie > 180 mg/dl besteht die Indikation für eine Insulintherapie.
- Ein Blutglukosezielbereich von 140–180 mg/dl ist für die meisten Patient*innen anzustreben.
- Ausgewählte Patient*innen können von einer strengeren glykämischen Kontrolle mit einer Blutglukose von 110–140 mg/dl profitieren, wenn diese ohne signifikante Hypoglykämien erreicht werden kann.
- Für die Entscheidung über mögliche Modifizierungen der antidiabetischen Therapie während eines Krankenhausaufenthaltes sind standardisierte Blutglukosezielwerte notwendig.
- Die Blutzuckerzielwerte sind individuell je nach Komorbiditäten, Begleitmedikation, Ernährungsstatus und Aufnahmegrund festzulegen. Durch eine strikte Blutzuckerkontrolle (Blutglukoseziel: 80–110 mg/dl vs. 180–200 mg/dl) konnte in der Leuven-Studie auf einer chirurgischen Intensivstation eine Reduktion der Mortalität erreicht werden. Ein ähnlicher Ansatz führte jedoch in der NICE-SUGAR-Studie sogar zu einer höheren Mortalität in der Patient*innengruppe mit niedrigeren Blutzuckerzielwerten [42]. Heterogene Patient*innenkollektive und Therapieschemata sind diesbezüglich nach wie vor für inkonklusive Empfehlungen verantwortlich. Eine Metaanalyse zeigte beispielsweise eine erhöhte Mortalität bei hospitalisierten Patient*innen, bei welchen die Blutzuckereinstellung zu strikt gewählt wurde [43].

### Nicht kritisch kranke Patient*innen

- Die Evidenz für einen eng definierten Blutglukosezielbereich nicht kritisch kranker Patient*innen ist nur eingeschränkt vorhanden, daher musste man sich bei der Definition von Zielbereichen für die Normalstation an die Empfehlungen aus dem intensivmedizinischen Bereich anlehnen.
- Ab einer Nüchternblutglukose > 140 mg/dl sollte eine Evaluierung von Ernährung und antidiabetischer Medikation erfolgen.
- Bei persistierender Blutglukose > 180 mg/dl besteht bei hospitalisierten Patient*innen die Indikation für eine Insulintherapie. Unter laufender Insulintherapie wird für den Großteil nicht kritisch kranker Patient*innen ein Blutglukosezielbereich von 140–180 mg/dl angestrebt.
- Bei strengeren Blutglukosezielbereichen (110-140 mg/dl) ist auf eine Vermeidung von signifikanten Hypoglykämien zu achten [44].
- Hypoglykämien < 70 mg/dl sollten unter stationären Bedingungen detektiert und dokumentiert werden, und etwaige Therapieadaptierungen sind durchzuführen [45].
- Bei terminal kranken Patient*innen mit schweren Begleiterkrankungen kann ein individuell höherer Blutglukosezielbereich festgelegt werden.
  Für die Erreichung der Therapieziele im Krankenhaus sind im Vergleich zur Therapieevaluierung zu Hause meist engmaschigere Blutglukosekontrollen notwendig. Bei guter und stabiler Blutglukoseeinstellung auch unter stationären Verhältnissen können die Empfehlungen aus dem Kapitel „Blutglukoseselbstkontrolle“ herangezogen werden. Eine Kontrolle der Blutglukose vor den Mahlzeiten sollte erfolgen. Wenn Patient*innen nicht essen, ist eine Blutglukosemessung zumindest alle 4–6 h durchzuführen [46]. Bei ausgeprägten Hyperglykämien, Hypoglykämien oder hoher glykämischer Variabilität ist meist zumindest ein 7‑Punkt-Profil indiziert. Eine intravenöse Insulintherapie ist alle 30–120 min mittels Blutglukosemessung zu evaluieren.

### Perioperative Empfehlungen

- Bei akuten Blutzuckerentgleisungen mit Hyperglykämien > 300 mg/dl sollten elektive Operation postponiert werden, bis sich die Blutglukose zumindest für 1–4 h präoperativ im Zielbereich (zumindest < 180 mg/dl) befindet [27, 29, 30].
- Die perioperative Stoffwechselkontrolle sollte primär mittels kapillärer Blutzucker- oder venöser Plasmaglukosemessung monitorisiert werden [7].
- Die perioperative Messfrequenz ist abhängig von der Fähigkeit der Menschen im Selbstmanagement und der Vigilanz. Präoperativ sollte während Nüchternheitsphasen zumindest alle 2–4 h gemessen werden. Im Falle von intravenöser (i.v.) Insulinzufuhr empfiehlt sich eine Messung alle 30–120 min [47].
- Ziele der perioperativen Glukosekontrolle sind das strikte Vermeiden von Hypoglykämien und hyperglykämischer Stoffwechselentgleisungen, da diese mit erhöhter Komplikationsrate sowie längerer Krankenhausaufenthaltsdauer und gesteigerter Mortalität assoziiert sind [8, 47–53]. Eine stabile perioperative Einstellung des Blutzuckers ist relevant, um das peri- und postoperative Risiko zu minimieren [54, 55].
- Perioperativ sollten Glukosewerte zwischen 110 und 140 mg/dl angestrebt werden, wobei ein Zielbereich von 80–180 mg/dl ebenso als adäquat anzusehen ist [21, 47, 48, 56, 57].

## Insulintherapie bei hospitalisierten Patient*innen

Ein großer Teil der Krankenhausaufenthalte von Patient*innen mit Diabetes mellitus erfolgt nicht wegen der Diabeteseinstellung per se, sondern aufgrund von Komorbiditäten bzw. anderen Ursachen. Eine Folge davon ist, dass während des stationären Aufenthaltes nur wenig Fokus auf der Qualität der Blutzuckerkontrolle liegt, speziell wenn sich Patient*innen auf nichtinternistischen Abteilungen befinden [58].

Die Art der Diabeteserkrankung (Diabetes mellitus Typ 1, Diabetes mellitus Typ 2, „Maturity Onset Diabetes of the Young“ [MODY] etc.) sollte aus der Krankenakte klar ersichtlich sein, nicht zuletzt auch damit gravierende Fehler, wie z. B. das vollständige Absetzen/Pausieren einer Insulintherapie bei Patient*innen mit Diabetes mellitus Typ 1, vermieden werden können.

Das Auslassen von Insulindosen prä- oder perioperativ, insbesondere aus Angst vor Hypoglykämien, kann bei Patient*innen mit Diabetes mellitus Typ 1 schwerwiegende, potenziell lebensbedrohliche Komplikationen wie eine perioperative Ketoazidose verursachen. Daher muss bei diesem Kollektiv das vorrangige perioperative Ziel sein, eine durchgängig adäquate Basalinsulinzufuhr zu gewährleisten, die eine stabile Stoffwechsellage ermöglicht. Ein präoperativer Nüchternblutzucker im normoglykämen Zielbereich darf kein Grund für eine unzureichende Substitution des Basalinsulins sein. Sollte es Hinweise für eine präoperativ zu hohe Basalinsulindosis geben (anamnestisch gehäufte Hypoglykämien), kann eine Dosisreduktion am Vorabend oder am Tag der Operation sinnvoll sein [47, 59–61].

Ein aktueller HbA_1c_-Wert ist bei allen stationären Patient*innen mit Diabetes mellitus erforderlich, da der HbA_1c_-Wert auch der Unterscheidung dient, ob eine längerfristige hyperglykämische Situation besteht oder die Blutglukoseerhöhung auf eine akute Erkrankung zurückzuführen ist. Bei der Interpretation des HbA_1c_-Wertes ist zu berücksichtigen, dass dieser durch Anämien, Transfusionen von Erythrozytenkonzentraten sowie schwere Nieren- oder Lebererkrankungen verfälscht sein kann [62]. Präoperativ sollte ein HbA_1c_-Wert unter 8,0 % erreicht werden, jedoch an die individuellen Therapieziele angepasst sein [30, 47]. Operationen bei HbA_1c_-Werten von über 10,0 % sollten nur bei vitaler bzw. dringlicher Operationsindikation durchgeführt werden. Bei Patient*innen, bei denen ein HbA_1c_-Wert präoperativ unter 8,0 % nicht möglich erscheint, sollte die Blutglukose zumindest 1–4 h vor der Operation unter 180 mg/dl liegen, um postoperative Komplikationen zu minimieren [30].

Ein aktives Diabetesmanagement unter Einbeziehung der Fähigkeiten des Selbstmanagements der Patient*innen wird dringend empfohlen. Die Diabeteseinstellung sollte entsprechend den individuell vereinbarten Therapiezielen erfolgen.

Eine Insulintherapie ist aufgrund der Wirksamkeit, der Steuerbarkeit und der fehlenden Medikamenteninteraktionen der beste Weg, eine Hyperglykämie bei hospitalisierten Patient*innen, insbesondere bei kritisch kranken Patient*innen sowie perioperativ, zu behandeln, und ist daher das Mittel der Wahl.

## Subkutane Insulintherapie

Die subkutane Insulintherapie ist der bevorzugte Weg der Blutglukosesenkung bei nicht kritisch kranken hospitalisierten Patient*innen außerhalb von Überwachungs- und Intensivstationen. Dabei ist eine basalorientierte Insulintherapie mit zusätzlicher Gabe von Bolusinsulin bei Patient*innen mit regelmäßiger Nahrungsaufnahme zu bevorzugen [46, 63]. Obwohl eine Mischinsulintherapie mit 2‑mal täglicher Gabe ebenfalls verwendet werden kann, zeigte sich in Studien, dass es dabei zu einem höheren Hypoglykämierisiko kommt [64].

**Abb. 1:**
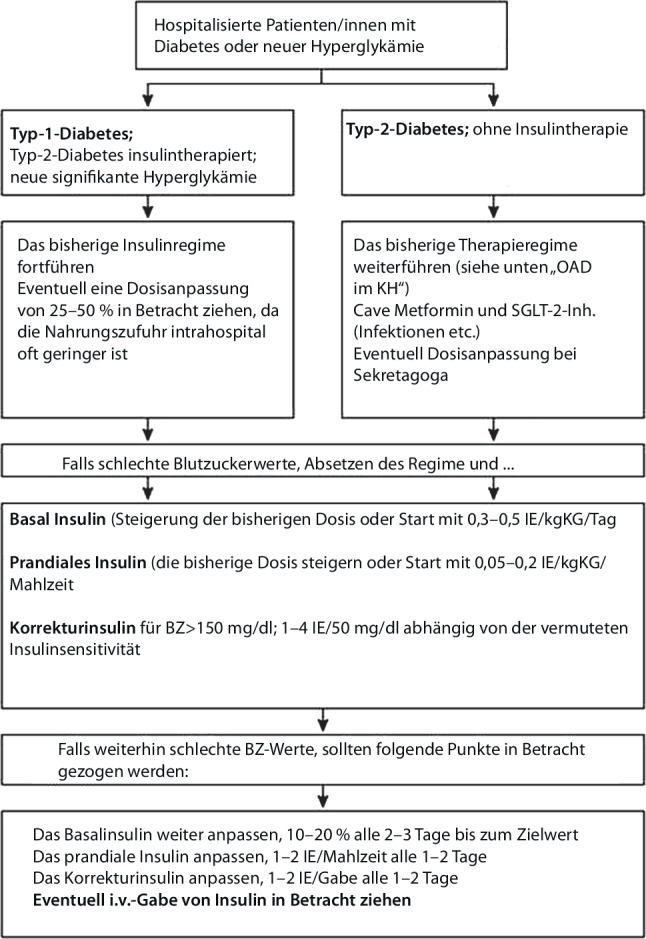
Algorithmus für das Blutzuckermanagement im intrahospitalen Bereich bei nicht kritisch kranken Patient*innen. (Adaptiert nach [69, 71]. Zur weiteren Therapie mit oralen/injizierbaren Antidiabetika s. weiter unten im Text)

Der initiale Insulintagesbedarf wird für die meisten Patient*innen auf ca. 0,3–0,5 IE/kg Körpergewicht geschätzt [65, 66]. Startdosen über 0,6–0,8 IE/kg Körpergewicht sind mit einem bis zu 3‑fach erhöhten Hypoglykämierisiko verbunden. Bei älteren Patient*innen (> 70 Jahre) und Patient*innen mit eingeschränkter Nierenfunktion verringert eine angepasste Startdosis von 0,2–0,3 IE/kg Körpergewicht das Hypoglykämierisiko [67]. Sollten Patient*innen nüchtern bleiben müssen (z. B. vor einer Operation) oder nehmen Patient*innen nur sehr kleine Mahlzeiten zu sich, ist es möglich, nur Korrekturinsulin zu verabreichen, sofern kein Typ-1-Diabetes besteht. Zu bevorzugen ist allerdings, dass auch eine stabile Basalinsulintherapie präoperativ nicht abgesetzt oder pausiert wird [68]. Ein möglicher Algorithmus wird in Abb. 1 dargestellt [69]. Zur Unterstützung bei der Insulindosistitration im stationären Bereich können Decision-Support-Systeme, sofern verfügbar, verwendet werden [70, 71].

## Management bei Insulintherapie im Krankenhaus

Die Durchführung unterschiedlicher Insulintherapieformen stellt v. a. auch für das Pflegepersonal eine große Herausforderung dar. Die folgende pragmatische Anleitung soll als Anhaltspunkt für die Kooperation zwischen den medizinischen Berufsgruppen dienen und Fehlanwendungen der Therapie verhindern.

### Grundregeln der Insulintherapie

- Lang wirksame (Basal‑)Insuline zum verordneten Zeitpunkt verabreichen. Diese Insuline werden typischerweise täglich (Neutrales Protein Hagedorn (NPH)-Insuline ggf. 2‑mal täglich) in derselben Dosierung und unabhängig vom aktuellen Blutzuckerwert verabreicht.
- Kurz wirksame oder ultrakurz wirksame (prandiale) Insuline immer unmittelbar vor der Mahlzeit applizieren. Diese werden nach aktuellem Blutglukosewert und Kohlenhydratgehalt der geplanten Mahlzeit dosiert.
- Mischinsuline immer unmittelbar vor der Mahlzeit des Verordnungszeitpunktes applizieren.
- Eine Umstellung einer Mischinsulintherapie ist bei Patient*innen, die nicht essen oder nüchtern bleiben müssen, sinnvoll. Hierzu empfiehlt es sich, die proportionalen Basalinsulindosen aus der Mischinsulinformulation herauszurechnen und (zumindest passager) auf eine Basis-Bolus-Insulintherapie umzustellen.
- Bei Hypoglykämien (Blutglukosewerte < 70 mg/dl) vor der Insulingabe rasche Korrektur der Hypoglykämie mit schnell wirksamen Kohlenhydraten und anschließend engmaschige Nachmessung der Blutglukose (cave: protrahierte oder rezidivierende Hypoglykämien). Nach Normalisierung der Glukose Verabreichung der für die nun folgende Mahlzeit festgesetzten Insulindosis (gegebenenfalls in reduzierter Dosis).

### Blutglukosemessungen – Wie oft und bei wem?

- In den ersten Tagen nach der Aufnahme bei bestehendem Diabetes mellitus, bei Neudiagnose eines Diabetes mellitus, Neueinstellung auf eine Insulintherapie oder Wechsel des Therapieschemas vor den Mahlzeiten sowie vor dem Zubettgehen
- 15 min nach einer Hypoglykämie und getroffenen Gegenmaßnahmen
- Bei stabilen Blutglukosewerten und einer Therapie mit oralen/injizierbaren Antidiabetika kann eine Reduktion der Messfrequenz auf 1‑ bis 2‑mal täglich nach einigen Tagen in Erwägung gezogen werden
- Bei Mischinsulintherapie – je nach Anordnung – auch 2 h nach der Mahlzeit
- Bei intensivierter Insulintherapie (Basalinsulin + kurz wirksames Insulin) Messungen optimalerweise vor den Mahlzeiten und 2 h danach
- Reduktion der Messfrequenz je nach Verlauf der Blutglukosewerte

## Intravenöse Insulintherapie

Kritisch kranke Patient*innen auf Intensiv- und Überwachungsstationen, aber auch Patient*innen mit diabetischer Ketoazidose (DKA) und/oder hyperglykämischen, hyperosmolaren Entgleisungen sollten primär mittels einer kontinuierlichen intravenösen Insulingabe behandelt werden [72, 73]. Klare Vorteile der intravenösen Insulingabe sind die bessere Steuerbarkeit, die raschere Möglichkeit, auf Entgleisungen zu reagieren, und die bessere Insulinwirkung mit einer kurzen Halbwertszeit durch eine intravenöse Applikation. In Österreich sind Human- und 4 Analoginsuline mit schnellem Wirkeintritt für die intravenöse Verabreichung zugelassen (Humaninsulin, Insulin aspart, Insulin glulisin, Insulin lispro, ultra-rapid Insulin lispro). Details sind im Kapitel zu den hyperglykämischen Entgleisungen zu finden.

Eine Umstellung der intravenösen Insulingabe auf eine subkutane Gabe sollte überlappend erfolgen. Nach der ersten subkutanen Gabe eines Basalinsulins sollte der Insulinperfusor noch für 2 h weitergeführt werden. Als subkutane Startdosis für das Basalinsulin werden 60 % der letzten, kumulativen intravenösen Tagesdosis (über die letzten 24 h) verwendet, wobei eine eventuell durchgeführte Umstellung der Ernährung (parenteral auf enteral) unbedingt bei der Abschätzung des Insulinbedarfs berücksichtigt werden muss. Bei Beginn der enteralen Ernährung sind die anderen 40 % der Tagesdosis gleichmäßig auf die Hauptmahlzeiten als Bolusinsulin aufzuteilen [72, 74]. Ein mögliches Protokoll wird in Tab. 1 dargestellt [75].

**Tab. 1:** Insulininfusionsschema. (Nach Goldberg et al. [75])

Blutglukosewert (mg/dl)	Insulindosis (ml/h = IE/h)
< 80 mg/dl	Perfusorpause und Kontrolle in 30 min
81–120 mg/dl	0,7 IE/h
121–150 mg/dl	1,0 IE/h
151–180 mg/dl	1,5 IE/h
181–210 mg/dl	2,0 IE/h
211–240 mg/dl	2,5 IE/h
241–270 mg/dl	3,0 IE/h
271–300 mg/dl	3,5 IE/h
301–330 mg/dl	4,0 IE/h
331–360 mg/dl	4,5 IE/h
361–390 mg/dl	5,0 IE/h
391–420 mg/dl	5,5 IE/h
421–450 mg/dl	6,0 IE/h

## Prä- bzw. intraoperatives Management

Eine Insulintherapie ist insbesondere bei schweren und längeren Eingriffen mit protrahierter intensivmedizinischer Betreuung derzeit die einzige therapeutische Option, perioperativ die Glukosewerte zu therapieren [76–78].

Bei allgemeinchirurgischen Patient*innen mit Diabetes mellitus Typ 2 zeigte die Gabe von Basalinsulin plus schnell wirksamem Insulin vor den Mahlzeiten (Basal-Bolus-Therapie oder Basal-Plus-Therapie) im Vergleich zu einer reaktiven Korrekturinsulingabe ohne Basalinsulin eine verbesserte glykämische Einstellung mit geringeren perioperativen Komplikationsraten [79, 80].

Bei großen Operationen mit protrahierter intensivmedizinischer Betreuung ist eine an aktuell gemessene Blutglukosewerte adaptierte i.v.-Verabreichung von kurz wirksamen Insulinanaloga die Therapie der Wahl. Empfehlenswert ist die gleichzeitige Bereitstellung von i.v.-Glukoseinfusionen (ggf. mit Kaliumzusatz), um hypoglykämische Werte rasch korrigieren zu können [81, 82].

Bei Patient*innen mit basalunterstützter oraler Therapie (BOT) kann bei Routineoperationen das abendliche bzw. morgendliche Basalinsulin in unveränderter Dosis appliziert werden, nachdem die orale Therapie präoperativ pausiert wurde. Engmaschige Blutglukosekontrollen perioperativ sind erforderlich, um allfällige Korrekturen mittels i.v.-Glukoseinfusion bzw. subkutaner zusätzlicher Gabe von rasch wirksamem Insulin bzw. Insulinanaloga zu gewährleisten. Eine Dosisreduktion des Basalinsulins um 20–25 % kann bei präoperativ eher niedrigen Glukosewerten sinnvoll sein [8, 47, 51, 83].

Patient*innen mit Basis-Bolus-Insulintherapie sollten bei Routineoperationen die vorgesehene Basalinsulindosis applizieren. Korrekturen der Blutglukosewertewerte erfolgen in Abhängigkeit von engmaschig durchgeführten Kontrollen mittels Glukoseinfusion oder kurz wirksamen Insulins [48, 81, 82]. Als Faustregel ist davon auszugehen, dass das Kohlenhydratäquivalent einer Kohlenhydrateinheit (KE = 10 g Kohlenhydrate [84]) den Blutglukosewert um 25–50 mg/dl hebt, eine zusätzlich gespritzte Einheit kurz wirksames Insulin den Blutzucker um 25–50 mg/dl senkt (in Abhängigkeit von, Körpergewicht, Insulinresistenz, Verteilungsvolumen und wirksamer Diabetestherapie). Somit müssen pro peroral oder intravenös zugeführter KE etwa 1–2 IE kurz wirksamen Insulins (prandiales Insulin) zusätzlich zum Basalinsulin verabreicht werden, um eine Normoglykämie zu gewährleisten.

Patient*innen unter Mischinsulintherapie sollten bei Routineoperationen auf ein lang wirksames Insulin (1- oder 2‑mal täglich verabreicht) umgestellt werden, wobei die zu veranschlagende Insulindosis des lang wirksamen Insulins etwa zwei Drittel der Standarddosis des ursprünglichen Mischinsulins betragen sollte. Entsprechende Korrekturen mit i.v.-Glukose und kurz wirksamem Insulin sind, wie oben dargestellt, durchzuführen [8].

Für die prä- bzw. intraoperative Phase wird folgendes Vorgehen empfohlen [85–87].

### Kurze Eingriffe

Subkutane Insulingaben können beibehalten werden, wenn durch die Operation nicht mehr als 1 bis 2 Mahlzeiten versäumt werden.

### Kleine Eingriffe am Morgen, durch die das Frühstück nur verzögert wird

Insulingabe verschieben, erst vor dem Frühstück applizieren.

Bei 1‑mal täglicher Gabe eines lang wirksamen Insulins: keine Änderung erforderlich, wenn die Dosis präoperativ adäquat war. Bei eher niedrigen präoperativen Nüchtern-Blutglukosewerten Dosisreduktion am Tag des Eingriffes (oder Vorabend je nach Applikationszeitpunkt) um 20–25 % erwägen.

### Wenn Frühstück und Mittagessen ausfallen

Kein kurz wirksames Insulin am Morgen.

### Lange und komplexe Eingriffe

In der Regel ist eine intravenöse Insulingabe notwendig.

Kontrollen der Blutglukose in < 1-stündlichem Abstand (häufiger bei Blutglukose < 100 mg/dl oder bei raschem Absinken der Blutglukose).

Engmaschige Elektrolytkontrollen für die Dauer der i.v.-Insulintherapie.

Eine Insulingabe ist bei Menschen mit Diabetes mellitus Typ 1 durchgehend erforderlich, um einer Ketoazidose und Hyperglykämie entgegenzuwirken. Der basale Insulinbedarf ist immer sicherzustellen. Eine alleinige Korrekturinsulingabe ist inadäquat.

## Orale Antidiabetika/Inkretin-basierte Therapien im Krankenhaus

In den meisten Fällen erfolgt eine Spitalsaufnahme nicht zur Adaptierung einer oralen antidiabetischen Therapie/Inkretin-basierten Therapie. Daher sollte man bei einer akuten Krankenhausaufnahme eine besser steuerbare passagere Insulintherapie andenken [44]. Bei Umstellung von einer Insulintherapie auf die antidiabetische Vortherapie sollte die Heimtherapie, sofern diese zuvor zu einer guten Einstellung zu Hause geführt hatte, 1 bis 2 Tage vor der geplanten Entlassung wieder initiiert werden. Dies dient dazu, eine unzureichende Diabeteseinstellung zu erkennen und die Diabetestherapie noch vor der Entlassung weiter anzupassen. Bei nicht kritisch kranken Patient*innen, kurzen Krankenhausaufenthalten, fehlenden Kontraindikationen und keiner akuten Stoffwechselentgleisung kann die antidiabetische Heimtherapie auch beibehalten werden [88, 89].

Die Initiierung einer oralen antidiabetischen Therapie/Inkretin-basierten Therapie zur Behandlung vor allem einer Stresshyperglykämie bzw. akuter Erkrankung wird bei hospitalisierten Patient*innen nicht empfohlen [44, 90]. Je nach klinischem Verlauf kann dann eine derartige Therapie eingeleitet werden.

Prinzipiell können alle oralen Antidiabetika/Inkretin-basierte Therapien auch bei hospitalisierten, nicht kritisch kranken Patient*innen weiterverwendet werden. Hervorzuheben ist jedoch die Bedeutung der Vermeidung von Hypoglykämien bei Beibehaltung der vorbestehenden, häuslichen Therapie sowie die Kontrolle von Leber- und Nierenfunktion aufgrund häufig akut einsetzender Beeinträchtigungen der Eliminationsorgane Leber und Niere.

Jedoch hat jede Substanzklasse bestimmte Einschränkungen, die im Folgenden aufgezählt werden.

### Perioperative medikamentöse Diabetestherapie

Generell sollten orale Antidiabetika am Tag der Operation (zumeist morgens) pausiert werden. Bei kurzen operativen Eingriffen kann die orale Therapie nach unkompliziertem chirurgischem Verlauf und Aufnahme der Nahrungszufuhr wieder eingeleitet werden. Bei längeren Operationen sollte frühestens am ersten postoperativen Tag die orale antihyperglykäme Therapie zeitgleich mit der ersten oralen Nahrungszufuhr wieder begonnen werden. Die folgenden Empfehlungen beziehen sich auf Menschen mit Diabetes mellitus und die entsprechende Indikation der genannten Substanzklassen, nicht aber auf weitere Anwendungsbereiche außerhalb der antihyperglykämen Wirkung (insbesondere Glukagon like peptide-1 Rezeptoragonisten (GLP-1-RA) und Sodium glucose linked transporter -2 (SGLT-2)-Inhibitoren).

## Metformin

### Metformin und Kontrastmittelgabe

#### Iodhaltige Kontrastmittel

Bei iodhaltigen Kontrastmitteln ist folgende Vorgehensweise empfohlen [91]:

1. Patient*innen mit einer eGFR > 30 ml/min/1,73 m^2^ und ohne Hinweise auf ein akutes Nierenversagen (AKI), die entweder ein intravenöses Kontrastmittel oder ein intraarterielles Kontrastmittel mit Second-Pass-Nierenexposition erhalten: Metformin wie gewohnt weiter einnehmen.
2. Patient*innen
  - mit einer eGFR < 30 ml/min/1,73 m^2^, die ein intravenöses Kontrastmittel oder ein intraarterielles Kontrastmittel mit Second-Pass-Nierenexposition erhalten,
  - die ein intraarterielles Kontrastmittel mit First-Pass-Nierenexposition erhalten,
  - mit AKI
    Metformin ab dem Zeitpunkt der Kontrastmittelgabe absetzen. eGFR innerhalb von 48 h kontrollieren und Metformin wieder beginnen, wenn sich die Nierenfunktion nicht wesentlich verändert hat.
3. Metformin ist im Notfall keine Kontraindikation für notwendige Untersuchungen. Um das Risiko einer Laktatazidose zu reduzieren, sollte nach der Untersuchung Metformin jedenfalls 48 h pausiert werden.

#### Gadolinium-basierte Kontrastmittel

Bei Gadolinium-basierten Kontrastmitteln sind keine speziellen Vorkehrungen notwendig [91, 92].

#### Metformin und Niereninsuffizienz

Metformin darf bei eingeschränkter Nierenfunktion bis zu einer eGFR von 30 ml/min/1,73 m^2^ bei fehlenden anderen Risikofaktoren für eine Laktatazidose eingesetzt werden [93]. Allerdings sollte es bei Patient*innen mit einer eGFR von 30–45 ml/min/1,73 m^2^ in reduzierter Dosis mit maximal 1000 mg täglich verwendet werden.

#### Metformin-assoziierte Laktatazidose

Die Inzidenz der Metformin-assoziierten Laktatazidose (MALA) wird mit 3 bis 10/100.000 Patient*innenjahre angegeben [94, 95].

Die Ursache einer MALA ist bis heute nicht restlos geklärt. Am Anfang dürfte ein plötzlicher rascher Anstieg der Metformin-Konzentration im Blut stehen, welcher bei eingeschränkter Leberfunktion eine Laktatproduktion triggern kann. Zur Akkumulation von Laktat und Metformin, welche dann zu einer Laktatazidose führen, kommt es beim Vorliegen bestimmter Begleitumstände wie einem akuten Nierenversagen, Hypovolämie, Sepsis, Herz-Kreislauf-Versagen, Alkoholismus, Leberzirrhose und anderen hypoxischen Zuständen (z. B. Schock).

An eine MALA sollte bei unspezifischen abdominellen Beschwerden in Verbindung mit Muskelkrämpfen gedacht werden. Eine Blutgasanalyse bestätigt das Ergebnis bei vermindertem pH-Wert und erhöhten Laktatspiegeln (> 5,0 mmol/l) [96].

Neben dem Absetzen von Metformin steht die Behandlung der Grundkrankheit im Vordergrund. Da Metformin nicht an Albumin gebunden ist, kann es durch eine Hämodialyse eliminiert werden. Allerdings hängt die Prognose der Patient*innen nicht von der Höhe der Metformin-Konzentration ab, sodass die Indikation zur Hämodialyse eher aufgrund eines evtl. auch bestehenden Nierenversagens gestellt wird.

#### Metformin und Erkrankungen

Bei Patient*innen, die aufgrund von schweren Infektionen, dekompensierter oder instabiler Herzinsuffizienz, Leberversagen oder auch schwerer Diarrhö und Exsikkose hospitalisiert wurden, muss Metformin pausiert werden.

#### Metformin und Operation

Für Metformin wird ein Absetzen am Tag der Operation mit Anästhesie (Allgemein‑, Spinal- oder Epiduralnarkose) empfohlen, wobei eine allfällige Kumulation von Metformin aufgrund von Nierenfunktionseinschränkung bzw. Nierenversagen zu verhindern ist (s. oben). Bei chronischer Nierenerkrankung mit Akkumulationsgefahr sollte die Einnahme präoperativ 24–48 h pausiert werden. Vor Wiederbeginn muss eine stabile und suffiziente Nierenfunktion (glomeruläre Filtrationsrate [GFR] > 30 ml/min) laborchemisch bestätigt werden, und entweder müssen 48 h postoperativ vergangen oder eine orale Nahrungsaufnahme muss möglich sein [97]. Bei sonst gesunden Menschen mit Diabetes genügt vor kleineren Eingriffen ein Pausieren von Metformin am Operationstag [47, 76, 77]. Jedenfalls sollte Metformin postoperativ bis zur Sicherstellung einer adäquaten Nierenfunktion pausiert bleiben.

## Pioglitazon

Pioglitazon darf nicht bei Patient*innen mit jeglicher Herzinsuffizienz (durch erhöhte Natriumrückresorption kommt es zu einer Flüssigkeitsretention) und bei eingeschränkter Leberfunktion (ALT > 2,5 × der Obergrenze des Normbereichs) angewandt werden.

### Pioglitazon und Operation

Aufgrund von mangelnder Evidenz wird das Pausieren von Glitazonen am Operationstag empfohlen [60].

## Sulfonylharnstoffe und Glinide

Aufgrund ihres Wirkmechanismus kann es unter Sulfonylharnstofftherapie zu Hypoglykämien kommen. Bei eingeschränkter Nierenfunktion kann die Akkumulation v. a. der lang wirksamen Sulfonylharnstoffe zu Hypoglykämien führen. Diese sollten daher bei hospitalisierten Patient*innen mit Vorsicht angewandt werden. Eine Studie zeigte mehr Hypoglykämien bei hospitalisierten Patient*innen unter Sulfonylharnstoffen verglichen mit Kontrollen [98]. Aber auch bei Patient*innen, die aufgrund einer interkurrenten Erkrankung im Krankenhaus weniger Nahrung zu sich nehmen, sollte die Sulfonylharnstofftherapie reduziert bzw. pausiert werden.

Bei Vorliegen einer Hyperglykämie unter Sulfonylharnstofftherapie bei hospitalisierten Patient*innen sind die Umstellung auf eine zumindest passagere Insulintherapie und das Pausieren/Absetzen des Sulfonylharnstoffs indiziert.

Bei eingeschränkter Leberfunktion kann durch die gestörte hepatische Glukoneogenese das Hypoglykämierisiko deutlich erhöht sein. Glinide werden vorwiegend hepatisch eliminiert und sind daher bei Patient*innen mit Leberversagen kontraindiziert.

### Sulfonylharnstoffe/Glinide und Operation

Sulfonylharnstoffe und Glinide können bei mangelnder Nahrungszufuhr (z. B. 12-stündige Nüchternphase präoperativ) Hypoglykämien auslösen. Zudem deuten tierexperimentelle Studien auf eine mögliche ungünstige Interferenz auf Hypoxie-bedingte Vasodilatation hin, was z. B. bei Patient*innen mit kritischer Koronardurchblutung Probleme verursachen könnte [99]. Es herrscht in der vorhandenen Literatur einheitlich die Empfehlung, Sekretagoga am Tag einer Operation zu pausieren bis eine orale Nahrungszufuhr wieder gewährleistet ist [60, 100–102].

## Dipeptidyl-Peptidase-4-Inhibitoren

Dipeptidyl-Peptidase-4(DPP-4)-Inhibitoren haben ein sehr geringes Hypoglykämierisiko und können daher auch bei eingeschränkter Nierenfunktion in adaptierter Dosis bei hospitalisierten Patient*innen verwendet werden. Einzig bei Linagliptin ist keine Dosisanpassung bei Niereninsuffizienz notwendig. Erste Studiendaten zeigen auch, dass DPP-4-Inhibitoren bei hospitalisierten Patient*innen gemeinsam mit einem Basalinsulin eine gleich gute Blutglukosesenkung erzielten wie Patient*innen mit einem lang und kurz wirksamen Insulin [103, 104]. Für Alogliptin und Saxagliptin besteht eine Warnung der FDA bezüglich Herzinsuffizienz [105]. Beide Substanzen sollten daher bei hospitalisierten Patient*innen mit Herzinsuffizienz nicht eingesetzt werden. Außerdem sollte bei Patient*innen mit nichtbiliärer Pankreatitis in der Anamnese oder chronischer Pankreatitis eine DPP-4-Inhibitortherapie nicht eingeleitet werden bzw. die Therapie bei Pankreatitisanamnese langfristig abgesetzt werden.

### Dipeptidyl-Peptidase-4-Inhibitoren und Operation

Aufgrund uneinheitlicher und unzureichender Evidenz im perioperativen Setting wird ein Pausieren am Tag der Operation für DPP-4-Inhibitortherapie empfohlen [47, 101, 103, 106–110].

## GLP-1-Rezeptoragonisten

GLP-1-Rezeptoragonisten (GLP-1-RA) haben ein sehr geringes intrinsisches Hypoglykämierisiko. Aufgrund positiver Endpunktdaten für Liraglutide, Semaglutide und Dulaglutide (LEADER-Studie, SUSTAIN-6-Studie, REWIND-Studie, SOUL-Studie, FLOW-Studie) sollte eine bestehende GLP-1-RA-Therapie gerade bei kardiovaskulär kranken Patient*innen, aber auch bei Patient*innen mit chronischer Nierenerkrankung möglichst nicht beendet werden [111–114].

Ausgenommen davon sind Aufnahmen aufgrund gastrointestinaler Ursachen (z. B. Übelkeit, Erbrechen etc.) und/oder akuter Pankreatitiden. Es ist zu berücksichtigen, dass GLP-1-RA zu einer verzögerten Magenentleerung und folglich zu gastrointestinalen Beschwerden führen können. Eine routinemäßige Kontrolle der Pankreasenzyme bei fehlender Klinik einer Pankreaserkrankung wird nicht empfohlen.

Im Zusammenhang mit Narkosen ist Vorsicht geboten, da eine verzögerte Magenentleerung das Risiko für Komplikationen während der Anästhesie erhöhen kann. Unter Verwendung von GLP-1-RA ist auch die Durchführung einer Ösophagogastroduodenoskopie mit einem erhöhten Risiko für die Retention von Nahrung und Aspirationen verbunden. Zudem scheint es einen Zusammenhang zwischen GLP-1-RA-Einnahme und einer unzureichenden Darmvorbereitung zu geben [115]. Studiendaten zeigen, dass Komplikationen vor allem während der Aufdosierungsphase auftreten, daher kann man überlegen, erst nach einem etwaig geplanten Eingriff eine GLP-1-RA-Therapie zu etablieren. Ein Absetzen der Therapie vor dem Eingriff (zumindest eine Woche bei 1‑mal wöchentlich zu applizierenden Substanzen) ist eine Option, eine weitere Option ist eine Verlängerung des Nüchternintervalls vor dem Eingriff. Im Zweifel kann vor dem Eingriff eine Sonographie des Magens erfolgen [116–118].

### GIP/GLP-1-Rezeptoragonisten

Der derzeit einzige zugelassene GIP/GLP-1-Rezeptoragonist Tirzepatid hat ein sehr geringes intrinsisches Hypoglykämierisiko. Eine kardiovaskuläre Endpunktstudie (SURPASS CVOT) zeigt einen kardiovaskulären Benefit mit Nichtunterlegenheit im Vergleich zu Dulaglutid. Daher sollte eine GIP/GLP-1-Rezeptoragonisttherapie bei kardiovaskulär erkrankten Menschen mit Diabetes mellitus oder solchen mit einem hohen kardiovaskulären Risiko nicht beendet werden. Ausgenommen sind auch hier Aufnahmen aufgrund einer gastrointestinalen Erkrankung/Pankreatitis [116, 118–120].

Auch bei GIP/GLP-1-Rezeptoragonisten gelten dieselben Vorsichtsmaßnahmen bezüglich Narkosen wie bei GLP-1-RA (s. GLP-1-Rezeptoragonisten).

## SGLT-2-Inhibitoren

SGLT-2-Inhibitoren sind klassische Sick-day-off-Medikamente und sollten daher in vielen Fällen bei hospitalisierten Patient*innen pausiert werden. Dies gilt insbesondere vor Operationen, bei längeren Fastenperioden, bei Reduktion der Insulindosis, bei Infektionen, bei Volumendefizit oder auch bei interkurrenten, schwerwiegenden Erkrankungen, wenn Ketonkörper vorhanden sind [121, 122]. Andererseits sollen SGLT-2-Inhibitoren bei stationärer Aufnahme von Patient*innen mit Diabetes mellitus Typ 2 aufgrund akut dekompensierter Herzinsuffizienz oder aufgrund von frischem Myokardinfarkt mit erhöhtem Risiko für Herzinsuffizienz weitergeführt oder frühzeitig neu begonnen werden, falls der klinische Gesamtzustand ausreichend stabil erscheint [123, 124].

Generell haben SGLT-2-Inhibitoren ein geringes Hypoglykämierisiko, und das auch nur, wenn sie in Kombination mit Insulin und/oder Sulfonylharnstoffen/Gliniden eingesetzt werden. Eine seltene, aber potenziell lebensbedrohliche Nebenwirkung unter dieser Therapie ist die euglykämische Ketoazidose, die v. a. bei plötzlich erhöhtem Insulinbedarf oder Reduktionen/Absetzen bestehender Insulintherapien auftreten kann. Durch eine venöse Blutgasanalyse und die Ketonkörpermessung (kapillär bzw. venös, alternativ mittels Harnketonbestimmung) kann dies sehr einfach diagnostiziert werden. Diese Abklärung ist bei allen Patient*innen mit klinischen Symptomen einer Ketoazidose unter SGLT-2-Inhibitortherapie empfohlen.

### SGLT-2-Inhibitoren und Operation

Aufgrund pharmakodynamischer Effekte der SGLT-2-Inhibitoren (Gliflozine), kommt es zu einer gesteigerten Ketonreabsorption und iatrogen induzierter Glukosurie [125, 126]. In Kombination mit relativem oder absolutem Insulinmangel mit daraus resultierender gesteigerter Ketogenese kann es zur seltenen, aber potenziell lebensbedrohlichen Nebenwirkung der euglykämen diabetischen Ketoazidose (EDKA) kommen, die aufgrund des Fehlens typischer Symptome und potenzieller Abwesenheit von relevanter Hyperglykämie verzögert erkannt werden kann. Hierbei sind Triggerfaktoren wie Reduktion oder Absetzen von Insulintherapien, Infektionen, Nüchternheit und bariatrische sowie herzchirurgische Eingriffe besonders risikobehaftet [126–135].

SGLT-2-Inhibitoren haben eine Halbwertszeit von 8–16 h, weshalb ein präoperatives Pausieren von 3 bis 5 Tagen rational begründbar ist [133, 136–138]. Internationale Empfehlungen einschlägiger Fachgesellschaften sind aufgrund der vorliegenden Datenlage uneinheitlich, empfehlen jedoch meist eine präoperative Pause von 3 Tagen [47, 139–144]. Aus Sicht der Autor*innen sollten SGLT-2-Inhibitoren bei Menschen mit Diabetes mellitus Typ 2 und lang dauernden Operationen (> 2 h), mit erwartbarer postoperativer Nahrungskarenz, bei größeren Eingriffen in Allgemeinnarkose – insbesondere bei Herz-OPs – sowie bei perioperativer Insulindosisreduktion optimalerweise 72 h präoperativ pausiert werden. Ein Wiederansetzen darf postoperativ erst bei stabiler kardiovaskulärer und metabolischer Situation erfolgen. Auch bei kurz dauernden Eingriffen in Regionalanästhesie ist aus Sicherheitsgründen ein Pausieren der SGLT-2-Inhibitoren von zumindest 48 h präoperativ empfehlenswert [145–147]. Bei notfallmäßigen Operationen hingegen soll die SGLT-2-Inhibitoreinnahme so zeitnah als möglich pausiert und perioperativ sollen die metabolische Kontrolle und Möglichkeit der seltenen Nebenwirkung einer EDKA beachtet werden [101].

Jedenfalls sollte das perioperative Management der Gliflozine nach individuellem Ermessen in Abhängigkeit der Indikation (Diabetes mellitus und/oder Herzinsuffizienz und/oder chronische Nierenerkrankung), Diabeteseinstellung und Therapieregime (Insulintherapie vs. orale antidiabetische Therapie) sowie Operationsart und -schwere interdisziplinär mit dem Team der Anästhesie festgelegt werden.

## Glukokortikoid-induzierte Hyperglykämie

Glukokortikoide können sowohl über eine Zunahme der Insulinresistenz als auch einer Betazellfunktionsstörung zu einer Hyperglykämie führen [122].

Epidemiologische Studien zeigen, dass im Krankenhaussetting bis zu 86 % jener, die orale oder intravenöse Glukokortikoide erhalten, zumindest eine hyperglykämische Episode aufweisen [148]. Als Risikofaktoren für das Auftreten einer Hyperglykämie wurden ein Alter > 65 Jahre, ein erhöhter Body-Mass-Index (BMI), eine positive Familienanamnese für Diabetes mellitus, ein Prädiabetes, ein HbA_1c_ ≧ 6,0 % (42 mmol/mol) vor der Glukokortikoidtherapie oder auch eine hohe Glukokortikoiddosis identifiziert [149]. In verschiedenen Patient*innenpopulationen konnte eine Assoziation zwischen dem Auftreten einer Glukokortikoid-induzierten Hyperglykämie und dem Outcome der Patient*innen gezeigt werden [150, 151]. Bei Risikopatient*innen ist es sinnvoll, einen HbA_1c_-Wert vor Initiierung einer Glukokortikoidtherapie zu bestimmen, um abschätzen zu können, ob nach Beendigung der Glukokortikoidtherapie potenziell auch eine Diabetesremission eintritt. In vielen Fällen bleibt jedoch ein Diabetes mellitus trotz Beendigung der Glukokortikoidtherapie bestehen.

Ab einer Glukokortikoiddosis von 7,5–20 mg Prednisolon oder Äquivalent (Cushing-Schwelle) ist mit einem diabetogenen Potenzial zu rechnen und dementsprechend ist auch ein Blutzuckertagesprofil während des stationären Aufenthaltes bei der Einnahme dieser Substanzen sinnvoll. Eine Nüchternglukosemessung allein ist zum Monitoring nicht ausreichend, da die Blutglukosewerte meist über den Tag als Konsequenz der meist morgendlichen Glukokortikoidverabreichung ansteigen. Topische oder inhalative Steroide haben ein vernachlässigbares Potenzial, Blutglukosewerte zu beeinflussen [152].

Die Evidenz zur Sicherheit und Effektivität von oralen Antidiabetika bei Glukokortikoid-induzierter Hyperglykämie im Krankenhaus ist gering. Für DPP-4-Hemmer und Sulfonylharnstoffe gibt es (schwache) Empfehlungen, insbesondere bei nur milden Hyperglykämien [153]. In den meisten Fällen ist eine Insulintherapie die bevorzugte Maßnahme zur Blutglukosesenkung.

Nachdem intermediär wirksame Glukokortikoide wie (Methyl‑)Prednisolon eine Wirkspitze nach 4–8 h aufweisen, bietet sich eine Therapie mit NPH-Insulin an, welches zeitgleich zum Glukokortikoid verabreicht werden sollte (Tab. 2 zur Dosisempfehlung [154]). Für länger wirksame Glukokortikoide wie Dexamethason oder bei mehrmals täglichen Gaben bieten sich länger wirksame Basalinsuline (Insulin degludec, Insulin glargin, Insulin glargin U300) an. Bei höheren Glukokortikoiddosen können zusätzliche prandiale Insulinapplikationen notwendig sein. Liegt bereits ein Diabetes mellitus mit Insulintherapie vor der Glukokortikoidtherapie vor, so sollte die Insulindosis unter Glukokortikoiden um zumindest 20 % angehoben werden. Weitere Insulindosisanpassungen unter regelmäßigen Blutglukosekontrollen sind essenziell.

**Tab. 2:** Mögliche NPH-Insulindosierung je nach Prednisolonäquivalentdosis [154].

Prednisolonäquivalentdosis (mg)	Insulindosis (IE/kgKG)
≥ 40	0,4
30	0,3
20	0,3
10	0,1

Wichtig ist die simultane Anpassung der Insulintherapie an etwaige Veränderungen, insbesondere Reduktionen oder Beendigungen von Glukokortikoidtherapien [152].

## Anwendung von Diabetestechnologie im Krankenhaus und bei Operationen

Die kontinuierliche Glukosemessung (CGM) bietet den Vorteil, dass sie im Gegensatz zu kapillären Blutglukosemessungen ein kontinuierliches Signal mit Trends sichtbar macht und – abhängig vom System – auch akustisch vor Blutzuckerentgleisungen warnt. Aktuell ist keines der kommerziellen Systeme, welche die Glukose im subkutanen Gewebe messen, für die Anwendung im Krankenhaus oder perioperativ zugelassen. Die COVID-Pandemie hat die Fülle an wissenschaftlicher Evidenz, die es zur Effektivität und Sicherheit von CGM-Systemen bei hospitalisierten Patient*innen gibt, durchaus positiv beeinflusst, da das Remote-Monitoring von Glukosewerten zu einer Reduktion von Besuchen am Bett durch das Pflegepersonal führt und dieses damit vor Ansteckung potenziell übertragbarer Erkrankungen geschützt werden konnte [155]. Zudem ist insbesondere das subkutane Glukosemonitoring hilfreich in der Reduktion der Arbeitslast des Personals und erlaubt eine engmaschigere Glukosekontrolle, welche potenziell auch zu Verbesserungen der Blutzuckereinstellung und konsekutiv auch Patient*innen-Outcomes beitragen könnte. Dementsprechend wurden auch kürzlich Konsensus-Statements von der amerikanischen und britischen Diabetesgesellschaft zum Thema Diabetestechnologie im Krankenhaus veröffentlicht [156, 157].

Die Weiterverwendung von CGM-Systemen im Krankenhaus und perioperativ ist zu befürworten [47, 158]. Bei Zweifel an deren Genauigkeit sollten im Bedarfsfall kapilläre Kontrollmessungen erfolgen. Erhobene CGM-Werte sollten vom Pflegepersonal mit einem Vermerk, dass der Wert vom CGM-System stammt, dokumentiert werden. Die Alarme des CGM-Systems sollten aktiviert sein, wobei die Alarmgrenzen für das Krankenhaussetting nicht speziell definiert sind. Jeder Alarm (insbesondere ein Hypoglykämiealarm) sollte eine kapilläre Messung nach sich ziehen. POC-Messungen erlauben in stabilen glykämischen Phasen auch eine Kalibration des CGM-Systems (sofern das Gerät Kalibrationen zulässt/erfordert). Es sollte darauf geachtet werden, dass CGM-Sensoren nicht an Stellen gesetzt werden, die möglicherweise durch eine medizinische Intervention oder Operation genutzt werden müssen (z. B. kein Setzen eines Sensors am Bauch bei geplantem oder möglicherweise notwendigem Kaiserschnitt bei schwangeren Frauen), und die Sensorsetzstelle nicht von Hautveränderungen wie Ödemen oder Hämatomen betroffen ist. Das Selbstmanagement der Patient*innen sollte regelmäßig evaluiert und bei Bedenken vorübergehend ausgesetzt werden.

Sofern kein Einsatz von Magnetresonanztomographie geplant ist, sollte Menschen mit Diabetes mellitus die perioperative CGM-Fortführung empfohlen werden. Es sollte jedenfalls zwischen kritisch und nicht kritisch kranken Menschen differenziert werden, wobei bei Letzteren die perioperative Fortführung von CGM und Nutzung der kontinuierlichen Werte sowie Trendpfeile empfohlen werden, sofern mit POC-Testungen übereinstimmend. Das geplante Operationsdatum sollte nicht am ersten oder letzten Tag eines Sensorlebens liegen, um Messungenauigkeiten zu minimieren, und die Tragestelle sollte an das Operationsgebiet angepasst und dokumentiert sein. Jedenfalls sollte die Entscheidung über die Fortführung oder Pausierung geplant und präoperativ festgelegt werden [159, 160].

Die Weiterverwendung von CGM-Systemen im Krankenhaus kann mit rechtlichen Risiken verbunden sein, da diese Systeme für die Verwendung im Krankenhaus nicht zugelassen sind. Krankenhäuser sollten entsprechende interne Richtlinien und Schulungen etablieren, um die sichere und rechtskonforme Nutzung von CGM-Systemen zu gewährleisten [156, 157].

Neben den generell bekannten Faktoren (Zeitverzögerung des subkutanen Signals, Sensordrift, Notwendigkeit der regelmäßigen Kalibration, Kalibration unter stabiler Glykämie), welche die CGM-Genauigkeit beeinflussen, kann die Genauigkeit des subkutanen CGM-Signals insbesondere unter stationären und perioperativen Bedingungen durch bestimmte Faktoren (Harnsäurekonzentration, Dehydratation, Vasokonstriktion, Hypotonie, Hypothermie, Hypoxie, stark fallende Blutzuckerkonzentration, Diathermie, Perfusion, Lagerung) sowie Medikamente (Paracetamol, Maltose, Ascorbinsäure, Mannitol, Heparin, Salicylsäure, Hydroxyurea) zusätzlich beeinflusst werden [161–163].

Mittlerweile liegen einige Studien vor, welche die Genauigkeit von unterschiedlichen CGM-Systemen bei hospitalisierten Patient*innen in verschiedenen Krankenhaussettings untersucht haben. Die Mehrheit der Studien untersuchte subkutane CGM-Systeme, und die Genauigkeit der CGM-Werte zu Referenzwerten war vergleichbar mit Studien aus ambulanten Settings und wird in einer rezenten Metaanalyse (ICU) mit einer „median absolute relative difference“ von 13 % (6,6–20,6; 11–14,7) beziffert. 98,7 % der Werte lagen im Clark Error Grid in der Zone A, was bedeutet, dass sich die Werte, die sich in diesem Bereich befinden, innerhalb von ±20 % (oder ±15 mg/dl bei niedrigen Werten) des tatsächlichen Referenzwertes befinden und damit als klinisch akzeptabel eingestuft werden können. Die Metaanalyse berichtet über noch genauere Werte von intravaskulären und transdermalen CGM-Systemen, welche sich jedoch noch in Erprobung befinden und in Europa derzeit noch nicht im routinemäßigen Einsatz sind [164]. In einem Kollektiv nach Kardiochirurgie (*n* = 60, 26,7 % mit Diabetes mellitus) konnte eine akzeptable CGM-Genauigkeit bei kurzer Nachbeobachtung von 23 h gezeigt werden [165]. Verglichen mit einem intravaskulären Mikrodialyse-CGM zeigte ein subkutanes CGM im Vergleich zur Referenzmethode bei herzchirurgischen Patient*innen (*n* = 24, 25 % mit Diabetes mellitus) perioperativ wiederholt niedrigere Werte (mittlere absolute relative Differenz (MARD) 6,5 % unter intravaskulärem vs. 30,5 % unter subkutanem CGM) [166]. Eine rezente Arbeit zur Genauigkeit eines Real-time-CGM während und nach großen Operationen (*n* = 29, 62 % kardiochirurgische Eingriffe, HbA_1c_ 5,9 %) ergab eine akzeptable Messgenauigkeit intra- und postoperativ [167]. Neben der Überprüfung der Messgenauigkeit gibt es auch einige Studien zur klinischen Effektivität von CGM-Systemen im Krankenhaussetting. Eine rezente Metaanalyse, in welche Studien eingeschlossen wurden, welche die Diabeteseinstellung mittels CGM vs. POCT-basierten Glukosewerten untersuchte, zeigte eine Reduktion von Hypo- und Hyperglykämien bei vergleichbarer Insulindosis [168]. Insbesondere die kritische Phase der Nacht kann durch CGM besser abgebildet werden und möglicherweise sonst nicht erfasste Hypoglykämien darstellen [63]. Auch perioperativ kann die Erkennung von in punktuellen Messungen unbemerkten Hypoglykämien durch den Einsatz von CGM-System verbessert werden [169, 170].

Studien zeigen allerdings eine gute Übereinstimmung von Werten, die mittels kapillärer POC-Messung und CGM gemessen wurden [171]. Insbesondere die kritische Phase der Nacht könnte durch CGM besser dargestellt werden und möglicherweise sonst nicht erfasste Hypoglykämien darstellen [63]. Auch für isCGM konnten vergleichbare Ergebnisse gezeigt werden [172]. Aktuell liegen 2 randomisiert kontrollierte Studien zum CGM-Einsatz bei hospitalisierten Patient*innen vor. Diese zeigten eine Verbesserung der glykämischen Kontrolle anhand der Zeit im Zielbereich sowie eine Reduktion von Hypoglykämien [173].

Telemetrie per CGM, also die Abbildung von Sensorwerten auf patient*innenfernen Monitoren, könnte möglicherweise in Zukunft die stationäre Behandlung von Patient*innen mit Diabetes ebenso optimieren. Auch intravaskuläre Systeme könnten zukünftig das kontinuierliche glykämische Monitoring insbesondere auf Intensivstationen verbessern [174]. Die Abbildung des Glukosewertes am Monitor als „fünfter Vitalparameter“ ist Gegenstand weiterer Forschungsambitionen.

Ob die Verwendung von CGM-Systemen im Krankenhaus tatsächlich auch zu verbesserten harten Endpunkten (Reduktion von Komplikationen, kürzere Hospitalisierungsdauer etc.) beitragen kann, ist Gegenstand zukünftiger Forschung.

Hinsichtlich der Entfernung von Sensoren vor bildgebenden Untersuchungen ist hier dasselbe Vorgehen wie bei der Insulinpumpentherapie indiziert (Tab. 4).

## „Hybrid closed loop“ im Krankenhaus

### AID-Systeme („hybrid closed loop“) und Insulinpumpentherapie im Krankenhaus und bei Operationen

Mit der wachsenden Verbreitung von AID-Systemen („hybrid closed loop“) nimmt auch die Zahl an hospitalisierten Patient*innen mit dieser Therapieform zu. Die Anwendung von AID-Systemen bei hospitalisierten Patient*innen mit Typ-2-Diabetes auf der Normalstation zeigt eine bessere Glukoseeinstellung ohne erhöhtes Hypoglykämierisiko, höhere Zeit im Zielbereich und reduzierte Glukosevariabilität verglichen mit subkutaner Insulintherapie und sollte deshalb, sofern möglich, im Rahmen des stationären Aufenthaltes fortgeführt werden [175, 176]. Bezüglich Typ-1-Diabetes findet sich wenig Evidenz in der Literatur [177, 178].

Die folgenden Empfehlungen beziehen sich vor allem auf die in Österreich verfügbaren Systeme:

- Medtronic (Medtronic, Dublin, Irland) 670G/780G (Smart Guard-Auto-Modus),
- CamAPS FX/Ypsopump (Burgdorf, Schweiz),
- Omnipod Dash (Acton, MA, USA) (kein AID-System, aber häufig als „Do it yourself“-System im Einsatz).

Patient*innen, die trotz ihrer Hospitalisierung körperlich und kognitiv in der Lage sind, ihre Diabetestherapie selbstständig fortzusetzen, sollen dies auch während des stationären Aufenthalts tun. Allerdings ist es notwendig, dass die betreffenden Krankenhäuser definierte Regeln für deren Anwendung haben [179], dass auf die Selektion geeigneter Patient*innen geachtet wird und dass aktive Kommunikation zwischen Patient*innen und medizinischem Personal bezüglich der AID/Insulinpumpentherapie erfolgt, damit eine sichere Anwendung ohne Patient*innengefährdung gegeben ist [180–183].

Des Weiteren sollte nach Möglichkeit das Personal Erfahrung im Umgang mit AID-Systemen oder Insulinpumpen haben. Generell ist jedoch anzumerken, dass die Patient*innen selbst meist mehr Erfahrung im Umgang mit AID-Systemen/Insulinpumpen als das jeweilige betreuende Team auf der Bettenstation haben werden. Der Fokus sollte in dem Fall auf Selbstmanagement gelegt werden, welches auch zu einer größeren Patient*innenzufriedenheit führt [184, 185].

Wenn die AID/Insulinpumpentherapie unter stationären Bedingungen fortgesetzt wird, sollte das Diabetesteam des betreffenden Krankenhauses im Verlauf des Aufenthaltes hinzugezogen werden, um die Therapie zu evaluieren. Des Weiteren sollten Pumpentyp, Insulintyp und Pumpeneinstellungen dokumentiert werden. Auch Therapieempfehlungen bzw. Dosisänderungen (Basalrate, Bolusdosis, Korrekturfaktor, Blutglukosemessfrequenz, Glukosezielbereich) sollten dokumentiert werden. Ebenso sollte in zeitnahen Abständen überprüft werden, ob die Patient*innen weiterhin in der Lage sind, die Therapie selbst zu steuern [47, 186]. Die Patient*innen sind auf die Zielbereiche im Krankenhaus hinzuweisen (z. B. 100–180 mg/dl) und, wenn notwendig, diese in den Pumpeneinstellungen für den Zeitraum des stationären Aufenthalts anzupassen [187].

Während des stationären Aufenthalts ist sicherzustellen, dass ausreichend Bedarfsmaterial für die Insulinpumpentherapie vorhanden ist. Katheterwechsel haben in gewohnter Regelmäßigkeit zu erfolgen. Durch das nosokomiale Keimspektrum ist besonders auf Infektionen im Bereich der Setzstellen zu achten [187].

Sollte eine Fortsetzung der AID/Insulinpumpentherapie nicht möglich sein, muss je nach Gesundheitszustand eine Umstellung auf eine subkutane Basis-Bolus-Insulintherapie erfolgen oder im Fall einer akuten Verschlechterung (Intensivpflichtigkeit, größere Operation, Ketoazidose etc.) auf eine intravenöse Insulintherapie umgestellt werden. Die Tab. 3 fasst die Kontraindikationen zur Fortsetzung der AID-/Insulinpumpentherapie im Krankenhaus zusammen [188]. Die Basalrate muss bei subkutaner Insulintherapie entsprechend durch ein Basalinsulin ersetzt werden. Die Bolusinsulindosis kann entweder mit 1/6 der bisherigen Insulintagesdosis jeweils zu den 3 Hauptmahlzeiten angenommen werden oder errechnet nach Insulin/Kohlenhydrateinheiten-Verhältnis verabreicht werden. Ein Korrekturschema sollte zudem vorgegeben sein [189].

**Tab. 3:** Kontraindikationen für eine AID-/Insulinpumpentherapie unter stationären Bedingungen. (Nach Mader et al. [188])

Veränderter Bewusstseinszustand (außer bei kurzer Anästhesie)
Patient*in zeigt sich nicht in der Lage, die Pumpe adäquat zu bedienen
Intensivpflichtigkeit
Psychiatrische Erkrankung (z. B. schwere Depression und/oder Suizidalität), die ein Diabetes-Selbstmanagement unmöglich macht
Diabetische Ketoazidose oder hyperosmolarer hyperglykämischer Zustand
Unwillen der Patient*innen, die Insulinpumpentherapie fortzusetzen
Mangel an Insulinpumpenzubehör
Mangel an qualifiziertem Fachpersonal (Diabetolog*innen, Diabetesberater*innen)
Entscheidung aus medizinischen Gründen

Bei kleineren Operationen/Eingriffen bei denen nicht mehr als eine Mahlzeit ausgelassen wird (z. B. ÖGD, Koloskopie etc.) kann die AID/Insulinpumpentherapie fortgeführt werden. Bei großen bzw. lang dauernden (> 2 h) Operationen sollten Patient*innen unter AID/Insulinpumpentherapie perioperativ mittels einer i.v.-Insulininfusionstherapie behandelt werden. Bei kurz dauernden (< 2 h), elektiven Eingriffen ist eine Fortführung der AID/Insulinpumpentherapie denkbar [8, 190]. Die Steuerung der von Patient*innen benutzten Insulinpumpe kann für nicht versierte Personen komplex sein [159, 191]. In Abhängigkeit von der Schwere und Dauer des geplanten Eingriffs, Vigilanz der Patient*innen und potenziellen Flüssigkeitsshifts ist eine Fortführung der AID/Insulinpumpentherapie perioperativ denkbar. Eine klare Empfehlung zur Fortführung kann aus der derzeitigen Evidenzlage nicht abgeleitet werden, weshalb hier zu einer individuellen Entscheidung geraten wird [159, 190, 192].

Rezent erschienene Fallberichte sowie kleinere randomisiert kontrollierte Studien deuten jedenfalls auf sicheren perioperativen Einsatz mit im Vergleich zur Standardtherapie überlegener glykämischer Kontrolle auch bei größeren und länger dauernden Operationen hin, sodass eine perioperative AID-Fortführung in Rücksprache mit den behandelnden Anästhesist*innen erwogen werden sollte. Relevant sind jedenfalls eine klare präoperative Kommunikation und Festlegung des Prozederes [175, 193–195]. In einem anästhesiologischen Konsensuspapier wird die Reduktion von Hypoglykämien durch AID/Insulinpumpensysteme betont, und die Fortführung bei nicht kritisch kranken Menschen perioperativ sollte erwogen werden (abhängig von der geplanten Operation/Eingriff, Volumenshifts und Hämodynamik). Es sollte jedenfalls präoperativ ein neues Insulininfusionsset an einer Stelle abseits des Operationsgebiets gesetzt werden und ausreichend Material ins Krankenhaus mitgebracht werden [159].

Im Falle von kritisch kranken Patient*innen mit vorbestehender, häuslicher Nutzung von Insulinpumpentherapie oder AID-Systemen, bei erwartbaren großen hämodynamischen oder Volumenshifts perioperativ ist die Umstellung auf eine intravenöse Insulinzufuhr präoperativ empfohlen [159].

Es kann manchmal sinnvoll sein, während unklaren oder nicht vorhersehbaren Nüchternphasen ein höheres temporäres Glukoseziel, den Sportmodus oder die Ease-off-Funktion einzustellen. Bei Operationen ist auch auf die Verwendung eines Teflonkatheters zu achten (Stahlkatheter sind bei Anwendung von Diathermiekautern kontraindiziert), auch die richtige Positionierung des Insulinabgabesystems ist in diesem Fall wichtig. Es sollte vorab eine Therapiealternative (i.v./s.c.-Insulin) geplant werden [196]. Auch im Rahmen der Geburt kann die AID/Insulinpumpentherapie fortgeführt werden. Hier ist es wichtig, schon im Vorfeld auf die Kathetersetzstelle zu achten, sodass diese im Falle einer Sectio nicht im Operationsgebiet liegt (z. B. Flanken). Sollte sich die werdende Mutter während der Entbindung unsicher im Umgang mit der AID/Insulinpumpentherapie fühlen oder der Blutzucker nicht ausreichend kontrolliert sein, sollte alternativ eine intravenöse Insulintherapie begonnen werden. Nach der Entbindung kann die AID-Therapie fortgeführt bzw. wieder gestartet werden. Es sollte nicht vergessen werden, den persönlichen Glukosezielwert der Systeme anzupassen sowie den Kohlenhydratfaktor bzw. die basalen Infusionsraten wie vor der Schwangerschaft zu verwenden, um das Hypoglykämierisiko zu minimieren [196].

**Abb. 2:**
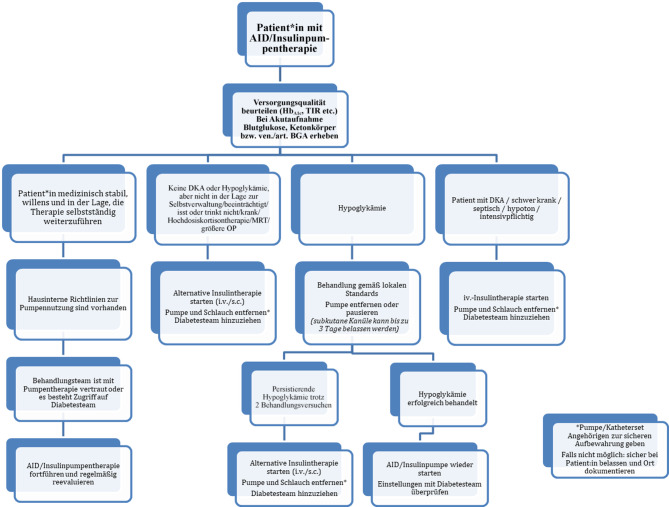
Empfehlungen für AID/Insulinpumpen im Krankenhaus. (Mod. nach Avari et al., Griffin et al. [187, 197])

Unter Glukokortikoidtherapie sollte eine engmaschige Überwachung der Glukoseverläufe sichergestellt sein. Grundsätzlich können die Algorithmen der AID-Systeme auf den erhöhten Insulinbedarf reagieren, ob dies jedoch ausreichend ist, hängt stark von Dosis und Therapiedauer ab. Vor allem bei höheren Glukokortikoiddosen kann es nötig sein, auf eine alternative Therapieform oder in den manuellen Pumpenmodus zu wechseln. Eine Anpassung (Senkung) des Kohlenhydratfaktors kann vor allem beim Frühstück/Mittagessen sinnvoll sein, um dem postprandial erhöhten Insulinbedarf Rechnung zu tragen. Ebenso kann eine Anpassung des temporären Ziels bzw. Nutzung der Boost-Funktion sinnvoll sein. Umgekehrt kann es beim Absetzen von Glukokortikoiden hilfreich sein, ein temporäres Ziel zu nutzen. Das Tapern von Glukokortikoiddosen kann in der Regel recht gut durch AID-Systeme bewältigt werden [197]. Im Rahmen der bildgebenden Diagnostik kann eine Unterbrechung der AID/Insulinpumpentherapie nötig sein. Für eine MRT-Untersuchung müssen die Insulinpumpe, Sensor und das Infusionsset entfernt werden. Insulinpumpe sowie Smartphone-App bei CamAPS FX und Infusionssets dürfen nicht in den MRT-Raum mitgenommen werden. Für CT-Untersuchung empfehlen die Hersteller das Abkoppeln der Pumpe vor der Aufnahme, bzw. diese mit der Bleiabdeckung abzudecken. Für Röntgenaufnahmen muss die Pumpe nicht entfernt werden, es sei denn, ihre Position beeinträchtigt die Bildgebung des relevanten Bereichs (Abb. 2). Der Umgang mit AID/Insulinpumpen während diagnostischer Verfahren ist in Tab. 4 dargestellt [197].

**Tab. 4:** Umgang mit der Insulinpumpe während diagnostischer Verfahren. (Nach Griffin et al. [197])

Röntgen/CT	Pumpe soll mit einer Bleischürze geschützt werden
MRT	Pumpe und Infusionsset aus Stahl müssen entfernt werden
Ultraschall	Pumpe kann an Ort und Stelle bleiben, der Schallkopf soll nicht direkt auf die Pumpe zusteuern
Herzkatheteruntersuchung	Pumpe soll mit einer Bleischürze geschützt werden
Schrittmacher‑/Defibrillator-Implantation	Pumpe soll mit einer Bleischürze geschützt werden
Koloskopie/Gastroskopie	Pumpe kann an Ort und Stelle bleiben
Laserchirurgie	Pumpe kann an Ort und Stelle bleiben

*CT* Computertomographie, *MRT* Magnetresonanztomographie

Vor der Entlassung aus einem Krankenhausaufenthalt sollte eine Konsultation des behandelnden Diabetesteams erfolgen, sodass Pumpeneinstellungen überprüft und an die geänderten Bedingungen nach dem Krankenhausaufenthalt angepasst werden können [189].

## Entscheidungsunterstützungssysteme für Diabetesmanagement im Krankenhaus

Bisher erfolgt in den meisten Fällen die Dokumentation von Blutglukosewerten manuell auf sog. „Diabeteskurven“. Auf diesen wird auch die jeweilige Insulindosis dokumentiert. Oft sind derartige Dokumente schwer leserlich geführt, es kann zu Übertragungsfehlern (Glukosewerte, Insulindosen) kommen, und trotz bestehender Guidelines werden Insulindosen aus Angst vor Hypoglykämien nur zögerlich gesteigert [198, 199]. Elektronische Diabetesmanagementsysteme mit integrierter Entscheidungsunterstützung können Blutglukosewerte direkt aus dem Laborinformationssystem importieren, grafisch darstellen und einen Insulindosisvorschlag für den jeweiligen Zeitpunkt geben [200]. Durch derartige Systeme wird das Diabetesmanagement besser visualisiert, und es kommt zu einer geringeren Fehlerhäufigkeit. Die Anwendung der Insulindosisvorschläge führt zu einer besseren Blutglukoseeinstellung auch während intrahospitaler Fastenphasen [201, 202]. Aktuell gibt es in Europa ein CE-zertifiziertes System (GlucoTab, decide Clinical Software GmbH), in den USA ist ebenfalls ein System (Glucommander, Glytec) von der Food and Drug Administration (FDA) zugelassen.

### Perioperatives Management – Zusammenfassung der Empfehlungen

1. Patient*innen mit Diabetes mellitus haben ein höheres Risiko für Begleiterkrankungen als gleichaltrige Patient*innen ohne Diabetes mellitus. Das Risiko für Multimorbidität steigt allgemein mit dem Lebensalter und insbesondere mit der Diabetesdauer. Zu erwartende Begleiterkrankungen betreffen insbesondere das kardiovaskuläre, renale System, die Nerven- und Sinnesorgane. Weiters bestehen häufig Zusatzerkrankungen im Sinne des metabolischen Syndroms.
2. Präoperative Untersuchungen sind in Abhängigkeit vom Umfang der geplanten Operation bzw. des Gesundheitsstatus der Patient*innen in enger Kooperation mit Anästhesist*innen und Chirurg*innen zu erheben.
3. Präoperativ sollte ein HbA_1c_-Wert unter 8 % vorliegen. Bei Patient*innen, bei welchen eine strikte Stoffwechselkontrolle nicht erzielbar bzw. aufgrund von begleitender Multimorbidität und fortgeschrittenem Alter nicht indiziert ist, sollte der HbA_1c_-Wert vor geplanten Operationen zumindest unter 8 % liegen. Operationen bei HbA_1c_-Werten von über 10 % sollten nur bei vitaler bzw. dringlicher Operationsindikation durchgeführt werden.
4. Generell sollten orale Antidiabetika am Tag der Operation (zumeist morgens) pausiert werden. Für Metformin wird bei Menschen mit Diabetes ohne Nierenfunktionseinschränkung ein Pausieren am Operationstag empfohlen, bei Nierenfunktionseinschränkung mit Akkumulationsgefahr 24–48 h präoperativ. SGLT-2-Inhibitoren bei Menschen mit Diabetes mellitus Typ 2 sollten vor größeren, lang dauernden Eingriffen in Allgemeinnarkose sowie bei perioperativer Insulindosisreduktion 72 h präoperativ, bei kurz dauernden Eingriffen in Regionalanästhesie zumindest 48 h und bei notfallmäßigen Operationen so zeitnah wie möglich pausiert werden. Ein Wiederbeginn der Therapie ist erst nach Stabilisierung der klinischen Akutsituation, Abklingen allfälliger Entzündungen und Normalisierung der Nierenfunktion zulässig. Sollte eine SGLT-2-Hemmer-Therapie „off-label“ bei Typ-1-Diabetes bestehen, ist diese im Setting um die Operation jedenfalls zu pausieren. Bei kurzen operativen Eingriffen kann die orale Therapie nach unkompliziertem chirurgischem Verlauf und Aufnahme der Nahrungszufuhr wieder angesetzt werden. Bei längeren Operationen sollte frühestens am ersten postoperativen Tag die orale antihyperglykämische Therapie wiederverordnet werden. Eine Kontrolle der Nierenfunktionsparameter vor neuerlicher Gabe von Metformin ist dabei erforderlich.
  Bei Inkretin-basierten Therapien (GLP-1-RA oder dualen Agonisten) ist eine präoperative Pausierung vorzunehmen oder eine verlängerte Nüchternperiode einzuhalten. Die Entscheidung des Fortführens oder Pausierens sollte interdisziplinär mit den behandelnden Anästhesist*innen getroffen werden.
5. Eine Insulintherapie ist perioperativ (vor allem bei schweren und längeren Eingriffen mit protrahierter intensivmedizinischer Betreuung) derzeit die einzige therapeutische Option, um Blutglukosewertewerte zu kontrollieren.
6. Ziele der perioperativen Glukosekontrolle sind das strikte Vermeiden von Hypoglykämien und ausgeprägter hyperglykämischer Stoffwechselentgleisungen mit Blutglukosewerten im Bereich von 80180 mg/dl. Bei kritisch Kranken (auf Intensivstationen) erfordern Blutglukosewerte > 180 mg/dl die Initialisierung einer kontinuierlichen, intravenösen Insulintherapie, unter welcher in weiterer Folge die Blutglukose zwischen 140 und 180 mg/dl gehalten werden sollte.
7. Auf Normalstationen sollten perioperativ Glukosewerte im Bereich von 80–180 mg/dl angestrebt werden. Blutzuckerwerte > 180 mg/dl sind zu vermeiden und bei Persistenz mittels Insulingabe zu therapieren.
8. Die Fortführung von kontinuierlichen Glukosemesssystemen (CGM) im perioperativen Management soll erwogen, jedoch durch POC-Testungen ergänzt werden. Potenziell interferierende Faktoren der CGM-Messgenauigkeit sowie die fehlende Zulassung in Österreich kommerziell erhältlicher Systeme für dieses Setting müssen beachtet werden. Ein Vorteil könnte bei Systemen mit Alarmfunktion in der Erkennung von bei punktuellen Messungen unerkannten Hypoglykämien liegen.
9. Bezüglich der perioperativen Insulinpumpentherapie oder AID-Systemen sollte immer in Abhängigkeit von der Schwere und Dauer des geplanten Eingriffs, Vigilanz der Patient*innen und potenziellen Flüssigkeitsverschiebungen entschieden werden. Bei großen bzw. lang dauernden (> 2 h) Operationen sollte auf eine intravenöse Insulininfusion umgestellt werden, während bei kurz dauernden (< 2 h), elektiven Eingriffen eine Fortführung der AID/Insulinpumpentherapie denkbar ist. Relevant ist jedenfalls eine klare präoperative Kommunikation mit den Teams der Anästhesie und Chirurgie und Festlegung des Prozederes.

## Interessenkonflikt

J.K. Mader gibt folgende Offenlegungen an: Advisory Boards von Abbott Diabetes Care, Becton-Dickinson, Biomea Fusion, Dexcom, Eli Lilly, Embecta, Insulet, Medtronic, myLife, Novo Nordisk A/S, Pharmasens, Roche Diabetes Care, Sanofi-Aventis, Tandem und Viatris; Honorare für Vortragstätigkeit: A. Menarini Diagnostics, Abbott Diabetes Care, Dexcom, Eli Lilly, Medtrust, MSD, Novo Nordisk A/S, Roche Diabetes Care, Sanofi, Sinocare, Viatris und Ypsomed. Gesellschafterin: decide Clinical Software GmbH und elyte Diagnostics. F. Aberer gibt folgende Offenlegungen an: erhielt Vortragshonorare von Sanofi Aventis, Novo Nordisk, AMGEN und Eli Lilly und war Mitglied in Advisory Boards von Eli Lilly und Novo Nordisk. J.M. Brix hat von folgenden Unternehmen Honorare für Vorträge, Beratertätigkeit sowie Forschungsunterstützung erhalten: Abbott, AstraZeneca, Bayer, Boehringer Ingelheim, Dexcom, Eli Lilly, Medtronic, Novo Nordisk. D.A. Hochfellner gibt folgende Offenlegungen an: Reisehonorare von Eli Lilly, Novo Nordisk, Novartis, MSD, Sanofi, Vortragstätigkeit Novo Nordisk und Sanofi. A.-T. Kietaibl gibt folgende Offenlegungen an: Advisory Board: Roche Diabetes Care Austria, Vortragstätigkeit: Sanofi-Aventis, Kongressunterstützung: Novo Nordisk, Eli Lilly, Boehringer Ingelheim. B. Ludvik gibt folgende Offenlegungen an: „Grants, research support and honorary for Advisory Boards and speaker’s bureau“: Amgen, AstraZeneca, Boehringer Ingelheim, Daiichi Sankyo, Eli Lilly, Madrigal, Novo Nordisk, Sanofi. M. Resl gibt folgende Offenlegungen an: Honorare für Vorträge und/oder Beratertätigkeit bzw. Fortbildungsunterstützung: Boehringer Ingelheim, Dexcom, Abbott, Eli Lilly. L. Stechemesser gibt folgende Offenlegungen an: Vortragstätigkeit und Forschungszusammenarbeit: Abbott, Amarin, Amgen, AstraZeneca, Bayer, Boehringer Ingelheim, CSL Vifor, Dexcom, Eli Lilly, Medtronic, Medtrust, Mylan, Novartis, Novo Nordisk, Roche Diabetes Care, Sanofi-Aventis. A. Vonbank: In den letzten 12 Monaten erhielt ich von folgenden Seiten Fortbildungsunterstützungen bzw. Honorare für Forschungstätigkeit, Beratungstätigkeit oder Vorträge: Eli Lilly, Boehringer Ingelheim, Amarin, Daiichi Sankyo, Novo Nordisk, Sanofi-Aventis, AstraZeneca, Amgen, Bayer, LKH Feldkirch. H. Sourij gibt folgende Offenlegungen an: Advisory Boards von Amgen, Amarin, Boehringer Ingelheim, Bayer, Eli Lilly, Daiichi Sankyo, Novartis, Novo Nordisk, Sanofi; M. Clodi hat von folgenden Unternehmen Honorare für Vorträge und Beratertätigkeit erhalten: Novo Nordisk, Elli Lilly, Boehringer Ingelheim, AstraZeneca,Novartis,Daiichi Sankyo, Amgen, Wörwag, Sanofi, Amarin, Roche, Bayer, Sobi. Honorare für Vortragstätigkeit: Amgen, Amarin, Boehringer Ingelheim, Bayer, Eli Lilly, Daiichi Sankyo, Novartis, Novo Nordisk, Sanofi. B. Föger und C. Ress-Winkel geben an, dass kein Interessenkonflikt besteht.
